# Clinical features of chronic tension-type headache combined with hyperacusis: a cross-sectional study

**DOI:** 10.1590/1516-3180.2025.3205.09032026

**Published:** 2026-06-01

**Authors:** Zenghui Fu, Yan Jin, Zaihong Lin, Yang Liu, Kedong Guo

**Affiliations:** I MSc. Lecturer, Physician, Department of Neurology, The Third Affiliated Hospital of Qiqihar Medical University, Qiqihar, China.; II MSc. Lecturer, Physician, Department of Neurology, The Third Affiliated Hospital of Qiqihar Medical University, Qiqihar, China.; III MSc. Associate Professor, Physician, Department of Neurology, The Third Affiliated Hospital of Qiqihar Medical University, Qiqihar, China.; IV BS. Nurse Practitioner, Department of Neurology, The Third Affiliated Hospital of Qiqihar Medical University, Qiqihar, China.; V MSc. Lecturer, Pathology School, Qiqihar Medical University, Qiqihar, China.

**Keywords:** Tension-type headache, Hyperacusis, Sleep quality, Anxiety, Cross-sectional studies, Pericranial tenderness, Loudness discomfort level, Sound intolerance, Chronic pain

## Abstract

**BACKGROUND:**

Chronic tension-type headache (CTTH) is a prevalent primary headache disorder that significantly affects quality of life. Hyperacusis, an abnormal intolerance to everyday sounds, is frequently observed in patients with CTTH, suggesting potential shared neural mechanisms. However, the clinical characteristics of patients with CTTH and comorbid hyperacusis remain poorly characterized.

**OBJECTIVES:**

This study aimed to investigate the clinical characteristics of CTTH patients with comorbid hyperacusis, focusing on headache profiles, auditory sensitivity patterns, and psychological correlates, and to identify factors independently associated with hyperacusis in this population.

**DESIGN AND SETTING:**

A cross-sectional study was conducted at the outpatient department of the Third Affiliated Hospital of Qiqihar Medical College from January 2022 to December 2024.

**METHODS:**

We consecutively enrolled 234 patients with CTTH diagnosed according to International Classification of Headache Disorders, 3rd edition (ICHD-3) criteria were consecutively enrolled. Hyperacusis was confirmed via otolaryngological evaluation and audiometric testing (pure-tone thresholds and loudness discomfort levels [LDL]). Demographic data, headache features (frequency, intensity, and duration), hyperacusis distress severity, and psychological measures (Generalized Anxiety Disorder-7 [GAD-7], Self-rating Somatic Symptom Scale [SSS], and Pittsburgh Sleep Quality Index [PSQI]) were analyzed. Univariate analyses (Student’s *t*-test and Chi-squared test) and multivariate logistic regression adjusted for potential confounders were performed to compare patients with (n = 47) and without (n = 187) hyperacusis.

**RESULTS:**

Hyperacusis prevalence was 20.09% (47/234). Compared to non-hyperacusis patients, hyperacusis patients exhibited longer headache duration (13.9 ± 3.15 versus 8.11 ± 2.74 years, *P* = 0.01), higher weekly headache frequency (7.30 ± 1.99 versus 4.19 ± 1.2, *P* = 0.005), more pericranial tenderness (95.74% versus 77.54%, *P* = 0.008), and poorer sleep quality (PSQI: 14.01 ± 1.13 versus 9.17 ± 0.51, *P* = 0.004). After adjusting for age, sex, headache duration, and pain intensity in multivariate logistic regression, higher headache frequency (odds ratio [OR] = 1.42, 95% confidence interval [CI]: 1.12-1.79, *P* = 0.003) and higher PSQI scores (odds ratios = 1.38, 95% CI: 1.15-1.66, *P* = 0.001) remained independently associated with hyperacusis. Common triggering sounds included children crying (61.7%) and traffic noise (42.55%), with predominant emotional responses being irritability (76.6%) and anxiety (65.96%). LDL classification (analyzed as an ordinal variable) was correlated with headache duration (*P* < 0.05), while hyperacusis distress severity was linked to headache frequency and PSQI scores (*P* < 0.05).

**CONCLUSION:**

CTTH with hyperacusis represents a distinct subgroup characterized by a history of prolonged headaches, increased attack frequency, and significant sleep disturbances. The independent associations identified suggest that sound sensitivity is closely linked to headache burden and sleep quality; however, causal relationships require longitudinal investigation. Integrated management addressing both headache and auditory hypersensitivity is warranted.

## INTRODUCTION

Chronic tension-type headache (CTTH) is one of the most prevalent primary headache disorders, characterized by frequent bilateral pressing pain that significantly impacts quality of life.^1^ Although the pathophysiology of CTTH remains incompletely understood, peripheral and central sensitization mechanisms are thought to play key roles.^2^ Notably, a subset of patients with CTTH experience comorbid hyperacusis-an abnormal intolerance to everyday sounds-suggesting potential shared neural mechanisms involving sensory processing dysregulation.^3^ However, the clinical features and implications of hyperacusis in patients with CTTH have not been systematically investigated.^4^

Emerging evidence suggests that hyperacusis may not merely be an accompanying symptom but rather may be associated with more severe central sensitization in headache disorders.^5^ Patients with coexisting CTTH and hyperacusis often report a greater headache burden, emotional distress, and functional impairment.^6^ However, critical questions remain unanswered: What distinguishes patients with CTTH and hyperacusis from those without? How does hyperacusis severity correlate with headache profiles? And what are the most troublesome sound triggers in this population? Addressing these questions could provide insights into phenotypic variability and guide targeted therapeutic strategies.^7^

This study aimed to comprehensively characterize patients with CTTH harboring hyperacusis by examining (1) demographic and headache profile differences between patients with and without hyperacusis; (2) auditory sensitivity patterns and their emotional impacts; and (3) relationships between hyperacusis measures and headache characteristics. Given the observational nature of this study, we focused on describing associations rather than inferring causal mechanisms. Our findings may help identify a clinically relevant CTTH subtype that requires multidisciplinary management approaches.^8^

## METHODS

### Participants

Consecutive patients diagnosed with CTTH who visited the outpatient department of the Third Affiliated Hospital of Qiqihar Medical College from January 2022 to December 2024 were included in this study. The diagnostic criteria for CTTH were based on the International Classification of Headache Disorders, 3rd edition (ICHD-3),^9^ demonstrating recurrent headache episodes lasting 30 min to 7 days, characterized by bilateral pressing or tightening pain of mild to moderate intensity, not aggravated by routine physical activity, and without nausea or vomiting (photophobia or phonophobia may be present). Additionally, headaches must occur on ≥ 15 days per month (≥ 180 days per year) for ≥ 3 months, meeting the essential features of tension-type headache while excluding medication-overuse headache.

All participants underwent medical history collection and routine otolaryngological examinations, with no history of otitis media or ear surgery, normal external ear canals and tympanic membranes, and with negative central nervous system physical examination results. Brain magnetic resonance imaging (MRI) ruled out intracranial and middle ear lesions, superior semicircular canal dehiscence, and brain tumors. Tympanometry revealed Type A curves, and stapedial reflexes were elicited with thresholds within the normal range.

Hyperacusis was diagnosed based on the following criteria:^10^ (1) patient-reported intolerance to everyday sounds not attributable to peripheral auditory pathology; (2) otolaryngological confirmation after excluding external, middle, and inner ear disorders; (3) mean loudness discomfort level (LDL) across tested frequencies ≤ 90 decibel hearing level (dB HL); and (4) symptoms are present for at least 3 months. Patients with a mean LDL > 90 dB HL who reported significant sound intolerance underwent a clinical interview to determine hyperacusis diagnosis based on functional impact.

The exclusion criteria were as follows: other primary or secondary headaches; medication overuse headache; history of head or neck trauma; systemic degenerative diseases such as rheumatoid arthritis or systemic lupus erythematosus; fibromyalgia syndrome; alcohol dependence or substance abuse; pregnancy; hyperacusis accompanied by tinnitus, where tinnitus was the most severe symptom affecting daily life; and severe psychiatric disorders.

All participants provided written informed consent before enrollment. The study was approved by the Ethics Committee of Qiqihar Medical College (2022-2037) and complied with the ethical requirements for clinical research in China and the Declaration of Helsinki.

### Collection of demographic and clinical data

Demographic and clinical data, including sex, age, height, weight, education level, smoking history, alcohol consumption history, diabetes, and hypertension, were collected. All participants maintained an 8-week headache diary to confirm their diagnosis and record headache clinical features. In the diary, patients recorded headache frequency (days per week), numeric pain rating scale (0 = no pain, 10 = worst pain),^11^ and duration of each headache episode (hours per day).

Participants confirmed by otolaryngologists to have hyperacusis were further assessed for the onset of hyperacusis, types of triggering sounds, emotional responses to triggering sounds, and the impact of hyperacusis. The degree of distress caused by hyperacusis was graded as follows:^12^ No distress: Not bothered at all. Mild distress: Slight discomfort, not particularly bothered by surrounding sounds, no impact on daily life. Moderate distress: Frequently aware of surrounding sounds, reduced concentration, occasional use of earplugs or avoidance of sounds, slight impact on daily life and work. Severe distress: Significant impact, frequent use of earplugs, avoidance of noisy environments, frequent irritability, moderate impact on daily life and work. Extreme distress: Severe impact, inability to tolerate any sound, fear of going outside, frequent anxiety or fear, near inability to maintain normal daily life, work, or study.

### Questionnaire assessments

The researchers involved in the study received training regarding the relevant assessment tools and selected appropriate times to administer the questionnaires. Questionnaires were distributed to all participants, collected on site, and immediately checked for completeness. If any omissions or issues affecting quality were found, participants were asked to promptly supplement or revise their responses.

1. Generalized Anxiety Disorder-7 (GAD-7):^13^ A Chinese-validated version with good reliability was used to screen and assess the severity of anxiety symptoms. The scale consists of seven items, each scored from 0-3, with higher scores indicating greater anxiety. A total score of 0-4 indicates no anxiety, 5-9 mild anxiety, 10-14 moderate anxiety, and ≥ 15 severe anxiety.
2. Self-rating Somatic Symptom Scale (SSS):^14^ A Chinese-validated version was used for self-assessment of somatic symptoms. The scale includes 20 items, each scored from 1-4, with higher scores indicating more severe somatic symptoms. Based on national norms, a total score ≥ 40 was considered positive for somatic symptom screening.
3. Pittsburgh Sleep Quality Index (PSQI):^15^ A Chinese-validated version was used to assess sleep quality. The scale consists of 18 items grouped into seven components: sleep latency, sleep frequency, sleep quality, sleep disturbances, sleep duration, sleep medication use, and daytime dysfunction. Total scores range from 0-21, with higher scores indicating worse sleep quality.

### Pure-tone audiometry and LDL testing

For CTTH patients with confirmed hyperacusis, pure-tone audiometry was performed using the Tinni Test^®^ tinnitus diagnostic and therapeutic system (Sichuan Micro-Digital Co., Ltd., Chengdu, China, model Tinni Test TTS-1000A). Air conduction thresholds were measured at 0.25, 0.5, 1, 2, 4, and 8 kHz. LDL testing was conducted using pure-tone stimuli at the same frequencies, starting at 70 dB above the threshold and adjusted in 5 dB steps until the patient reported discomfort. The mean LDL was calculated for each patient. Based on Goldstein’s classification,^16^ LDL was divided into four ordinal categories: Level 1 (≥ 95 dB at all frequencies), Level 2 (80-90 dB at ≥ 2 frequencies), Level 3 (65-75 dB at ≥ 2 frequencies), and Level 4 (≤ 60 dB at ≥ 2 frequencies).

### Statistical analysis

Statistical analyses were performed using GraphPad Prism v.6. Normally distributed continuous data are expressed as means ± standard deviation (SD) and compared using independent Student’s *t*-tests or analysis of variance (ANOVA). Categorical data are presented as counts and were compared using Chi-squared tests. To identify factors independently associated with hyperacusis, multivariate logistic regression analysis was performed with hyperacusis (present/absent) as the dependent variable. Independent variables included demographic factors (age, gender) and clinical variables that showed significant differences in univariate analyses (headache duration, headache frequency, pericranial tenderness, and PSQI scores). Variables were entered using forward stepwise selection with an entry criterion *P* < 0.05 and a removal criterion *P* > 0.1. Results are presented as odds ratios (ORs) with 95% CIs. Spearman’s rank correlation was used to assess relationships between variables, with LDL classification treated as an ordinal variable. Spearman’s ρ was calculated with distress levels treated as ordered categories for correlations involving hyperacusis distress severity. *P* < 0.05 was considered statistically significant.

## RESULTS

### Comparison of demographic and clinical characteristics between CTTH patients with and without hyperacusis

A total of 234 patients with CTTH were enrolled, of whom 47 (20.09%) had hyperacusis. Compared to patients without hyperacusis, those with hyperacusis had longer headache durations (*P* = 0.01), higher headache frequencies (*P* = 0.005), more frequent pericranial tenderness (*P* = 0.008), and higher PSQI scores (*P* = 0.004). No significant differences were observed in age, sex, body mass index, smoking, alcohol consumption, education level, diabetes, hypertension, headache pain intensity, headache duration, GAD-7 scores, or SSS scores (*P* > 0.05) (Table 1).

**Table 1.** Comparison of demographic and clinical characteristics between CTTH patients with and without hyperacusis.

Item	CTTH patients without hyperacusis (n = 187)	CTTH patients with hyperacusis (n = 47)	Test values	P values
Age (years, mean ± SD)	33.51 ± 11.4	32.56 ± 13.91	1.302	0.194
Female [n (%)]	112 (59.89)	30 (63.82)	0.107	0.734
BMI (kg/m², mean ± SD)	24.36 ± 2.51	23.97 ± 3.51	1.2	0.161
Smoking [n (%)]	19 (10.16)	4 (8.51)	0.004	0.948
Alcohol consumption [n (%)]	31 (16.58)	10 (21.28)	0.295	0.587
Duration of education (years, mean ± SD)	13.91 ± 3.51	14.56 ± 4.69	0.91	0.105
Diabetes [n (%)]	2 (1.07)	0 (0)	0.03	0.861
Hypertension [n (%)]	6 (18.38)	2 (20.51)	0.009	0.924
Headache duration (years, mean ± SD)	8.11 ± 2.74	13.90 ± 3.15	4.19	0.01
Headache pain score (mean ± SD)	5.19 ± 1.25	5.31 ± 1.68	1.301	0.069
Headache attack frequency (times/week, mean ± SD)	4.19 ± 1.2	7.30 ± 1.99	6.194	0.005
Headache duration (hours, mean ± SD)	6.17 ± 1.45	6.31 ± 1.73	1.71	0.061
Headache with pericranial tenderness [n (%)]	145 (77.54)	45 (95.74)	7.004	0.008
GAD-7 score (mean ± SD)	7.10 ± 1.69	7.19 ± 2.11	1.819	0.094
SSS score (mean ± SD)	8.18 ± 1.93	8.31 ± 1.7	1.104	0.18
PSQI score (mean ± SD)	9.17 ± 2.4	14.01 ± 3.91	6.194	0.004

Multivariate logistic regression analysis was performed to identify factors independently associated with hyperacusis. After adjusting for age, sex, headache duration, and pain intensity, two variables remained significantly associated with hyperacusis: higher headache frequency (OR = 1.42, 95% CI = 1.12-1.79, *P* = 0.003) and higher PSQI scores (OR = 1.38, 95% CI = 1.15-1.66, *P* = 0.001). Pericranial tenderness exhibited a trend toward significance (OR = 3.21, 95% CI = 0.98-10.52, *P* = 0.014). The model demonstrated good fit (Hosmer-Lemeshow χ² = 6.84, *P* = 0.554) (Table 2).

**Table 2.** Multivariate logistic regression analysis of factors independently associated with hyperacusis in CTTH patients.

Variable	β	S.E.	Wald χ²	OR	95% CI	P value
Headache frequency (times/week)	0.351	0.118	8.85	1.42	1.12-1.79	0.003
PSQI score	0.322	0.095	11.49	1.38	1.15-1.66	0.001
Pericranial tenderness (present vs. absent)	1.167	0.605	3.72	3.21	0.98-1.52	0.014
Constant	−5.873	1.402	17.55	0.003	-	< 0.001

The dependent variable was the presence of hyperacusis (coded as 1 for present and 0 for absent). Independent variables were entered as follows: pericranial tenderness (1 for present and 0 for absent), headache frequency (times per week; continuous), and PSQI score (continuous, 0-21). Variables included in the model were selected based on *P* values < 0.1 in univariate analyses and clinical relevance. A Hosmer-Lemeshow test *P* value > 0.05 indicated good model fit. Bold type indicates statistical significance (*P* < 0.05).

### Characteristics of hyperacusis

Among the 47 CTTH patients with hyperacusis, the mean duration of hyperacusis was 2.84 ± 0.31 years. The distribution of hyperacusis duration was as follows: < 6 months, 7 (14.89%); 7-12 months, 10 (21.28%); 1-5 years, 22 (46.81%); and ≥ 6 years, 8 (17.02%). Hyperacusis symptoms worsened progressively in eight (17.02%) patients, whereas 25 (53.19%) reported fluctuating symptoms. Additionally, 17 (36.17%) patients experienced ear fullness, pressure, itching, or echo sensations triggered by sounds, and 9 (19.15%) reported periauricular numbness or burning sensations.

Hearing was normal (speech-frequency pure-tone average ≤ 25 dB HL) in 35 (74.47%) patients, whereas 12 (25.53%) had unilateral sensorineural hearing loss (> 25 dB HL), with nine (19.15%) affecting the left ear and three (6.38%) the right ear. Based on hyperacusis distress severity, no distress: 4 (8.51%), mild distress: 17 (36.17%), moderate distress: 21 (44.68%), and severe distress: 5 (10.63%). The LDL classification (analyzed as an ordinal variable) was as follows: level 1 (≥ 95 dB at all frequencies): 8 (17.02%), level 2 (80-90 dB at ≥ 2 frequencies): 12 (25.53%), level 3 (65-75 dB at ≥ 2 frequencies): 12 (25.53%), and level 4 (≤ 60 dB at ≥ 2 frequencies): 10 (21.28%). Spearman’s correlation analysis revealed no significant relationship between the LDL classification and hyperacusis distress severity (ρ = 0.04, *P* > 0.05).

### Types of triggering sounds and emotional responses

The 47 patients reported discomfort to 10 types of sounds, most commonly: children crying/screaming: 29 (61.7%), crowded city noise: 26 (55.32%), traffic noise: 20 (42.55%), sudden high-pitched sounds: 18 (38.3%), loud laughter/cheering: 14 (29.79%), television sounds: 13 (27.66%). Each patient reported at least three negative emotional responses to triggering sounds, including: irritability: 36 (76.6%), anxiety: 31 (65.96%), reduced concentration: 20 (42.55%), and anger/irritability: 12 (25.53%).

### Correlation between hyperacusis and CTTH

Spearman’s correlation analysis was used to assess relationships between LDL classification (as an ordinal variable), hyperacusis distress severity, and CTTH clinical features (headache duration, frequency, pericranial tenderness, and PSQI scores). The results showed that LDL classification (ordinal) was positively correlated with headache duration and pericranial tenderness, and hyperacusis distress severity was correlated with headache frequency and PSQI scores (Table 3).

**Table 3.** Correlation between hyperacusis and CTTH.

	Headache durations	Pericranial tenderness	Headache frequency	PSQI scores
LDL classification	ρ = 0.612 *P* = 0.013	ρ = 0.854 *P* = 0.001	ρ = 0.266 *P* = 0.184	ρ = 0.195 *P* = 0.372
Hyperacusis distress severity	ρ = 0.304 *P* = 0.184	ρ = 0.269 *P* = 0.092	ρ = 0.704 *P* = 0.011	ρ = 0.761 *P* = 0.008

## DISCUSSION

The present study provides novel insights into the clinical characteristics of patients with CTTH harboring comorbid hyperacusis and revealed several important findings. First, our results demonstrate that approximately 20% of patients with CTTH exhibit hyperacusis, suggesting that this comorbidity is relatively common in this population. This prevalence rate is notably higher than that reported in the general population (estimated at 8-15%),^17^ suggesting a potential association between CTTH and auditory hypersensitivity.

The observed differences between patients with and without hyperacusis in the CTTH group are particularly noteworthy. Patients with hyperacusis had significantly longer headache duration, higher attack frequency, and more frequent pericranial tenderness, all of which are indicators of more severe and chronic disease. Multivariate analysis revealed that higher headache frequency and poorer sleep quality were independently associated with hyperacusis after adjusting for potential confounders. This suggests that these factors may represent key features of the CTTH-hyperacusis phenotype, rather than being mere correlates. These findings align with the concept of central sensitization, in which prolonged nociceptive input from pericranial muscles may lead to heightened sensitivity in both the trigeminal and auditory systems.^18^ However, given the cross-sectional design of this study, we cannot determine whether central sensitization precedes or results from the observed associations. Longitudinal studies are needed to establish temporal relationships. The strong association between pericranial tenderness and hyperacusis (95.74% versus 77.54%) further supports the possibility of a shared mechanism.^19^

Our study also revealed that hyperacusis in patients with CTTH is associated with significantly worse sleep quality, as evidenced by higher PSQI scores. The independent association between PSQI scores and hyperacusis in the multivariate analysis strengthens this finding. This finding is clinically relevant, as poor sleep is known to exacerbate pain sensitivity and may contribute to a vicious cycle of headache chronification.^20^ Notably, the direction of this association cannot be determined from our data; poor sleep may increase susceptibility to hyperacusis, hyperacusis may disrupt sleep, or both may share common underlying mechanisms. Interestingly, although anxiety and somatic symptom scores did not differ between the groups, the specific emotional responses to triggering sounds (notably irritability and anxiety in > 65% of patients) suggest that hyperacusis may represent a distinct dimension of psychological distress in CTTH patients.^21^ This apparent discrepancy may reflect the difference between state-like emotional responses to specific sound triggers versus trait-like anxiety as measured by the GAD-7 scaling.

The pattern of sound sensitivity observed in our cohort, particularly to high-pitched and sudden sounds, such as children crying (61.7%) and traffic noise (42.55%), mirrors the findings in migraine-related hyperacusis,^22^ suggesting possible commonalities in auditory processing abnormalities across primary headache disorders. However, the lack of correlation between LDL measures and subjective hyperacusis distress highlights the complex nature of sound intolerance, in which psychological factors may modulate perceptual sensitivity independent of pure physiological thresholds.^23^ This dissociation underscores the importance of assessing both objective auditory measures and subjective distress in clinical evaluations.

Several limitations should be considered. First, the cross-sectional study design precludes causal inferences concerning the relationship between CTTH and hyperacusis.^24^ Our findings represent associations that require confirmation in longitudinal studies to establish temporal sequences and potential causal pathways. Second, we did not assess other potential contributors to central sensitization, such as cutaneous allodynia.^25^ Third, the single-center nature of the study may limit its generalizability.^26^ Fourth, although multivariate analysis adjusted for several potential confounders, residual confounding from unmeasured variables (e.g., medication use, stress levels, and psychiatric comorbidities) cannot be excluded. Future longitudinal studies incorporating quantitative sensory testing and neuroimaging could help to elucidate the neural mechanisms underlying this comorbidity.^2^

From a clinical perspective, our findings suggest that assessment of sound sensitivity should be routinely included in the evaluation of patients with CTTH, particularly those with long-standing symptoms and pericranial tenderness.^27^ The identification of hyperacusis may help identify a distinct CTTH subgroup that could benefit from targeted interventions addressing both headache and auditory hypersensitivity, potentially including cognitive-behavioral approaches for sound intolerance alongside conventional headache therapies.^28^ However, interventional studies are needed to determine whether such integrated approaches improve outcomes in this patient population.

## CONCLUSION

This cross-sectional study demonstrated that approximately 20% of patients with CTTH present with comorbid hyperacusis, characterized by longer headache duration, higher attack frequency, more frequent pericranial tenderness, and significantly worse sleep quality compared to those without hyperacusis. Multivariate analysis identified higher headache frequency and poorer sleep quality as independent factors associated with hyperacusis in this population. The pattern of sound sensitivity predominantly involves high-pitched and sudden sounds, with irritability and anxiety being the most common emotional responses. These findings suggest that CTTH with hyperacusis represents a clinically distinct subgroup that requires integrated management strategies. Future longitudinal studies are warranted to establish causal relationships and evaluate the efficacy of targeted interventions addressing both headache and auditory hypersensitivity.
